# Modified nucleosides: an accurate tumour marker for clinical diagnosis of cancer, early detection and therapy control

**DOI:** 10.1038/sj.bjc.6603164

**Published:** 2006-05-09

**Authors:** A Seidel, S Brunner, P Seidel, G I Fritz, O Herbarth

**Affiliations:** 1 Institute of Hygiene, Faculty of Medicine, University of Leipzig, Liebigstrasse 27, D-04103 Leipzig, Germany; 2 Institute of Medical Biophysics and Physics, Faculty of Medicine, University of Leipzig, Liebigstrasse 27, D-04103 Leipzig, Germany; 3 Environmental Hygiene and Epidemiology (Environmental Medicine), Faculty of Medicine, University of Leipzig, Liebigstrasse 27, D-04103 Leipzig, Germany; 4 Department of Human Exposure Research and Epidemiology, UFZ – Centre for Environmental Research Leipzig-Halle, Permoserstrasse 15, D-04318 Leipzig, Germany; 5 Environmental Hygiene and Epidemiology (Environmental Medicine), Faculty of Medicine, University of Leipzig, Liebigstrasse 27, D-04103 Leipzig, Germany

**Keywords:** differential diagnosis, modified nucleosides, tumour marker, urine

## Abstract

Modified nucleosides, regarded as indicators for the whole-body turnover of RNAs, are excreted in abnormal amounts in the urine of patients with malignancies. To test their usefulness as tumour markers and to compare them with the conventional tumour markers, fractionated urine samples were analysed using chromatography. The excretion patterns of nucleosides of 68 cancer patients with malignant and benign tumours and 41 healthy controls have been studied. Significant elevations in the total sum and the concentrations of at least three (or four) of indicator nucleosides cytidine, pseudouridine, 2-pyridone-5-carboxamide-N1-ribofuranoside, N2,N2-dimethylguanine, 1-methylguanosine, 2-methylguanosine and 1-methyladenosine indicate a tumour with a sensitivity of 54% (77%) and a specificity of 86% (98%). Using an artificial neural network analysis, a sensitivity of 97% and a specificity of 85% were achieved in differentiating between tumour and control volunteers. The comparison with carcinoembryonic antigen, cancer antigen 15-3 und tissue polypeptide antigen indicates that urinary nucleosides may be useful tumour markers. This study suggests that the simultaneous determination of modified nucleosides and creatinine in urine samples of patients with cancer leads to an advantage to current methods and is a useful method to detect cancer early and to control the success of therapy.

Urinary excretion of modified nucleosides by healthy adults is relatively low, but is an indication of the whole-body turnover of ribonucleic acids (RNAs) (Sander *et al*, 1986; Schöch *et al*, 1990a, 1990b; Topp *et al*, 1993). Urinary excretion of methylated nucleosides, which results from increased turnover and degradation of RNA, especially from transfer ribonucleic acid (tRNA), has been shown to be excreted in increased amounts in the urine of patients with different types of tumours and AIDS (Nakano *et al*, 1993; Xu *et al*, 2000; Yang *et al*, 2004; Zheng *et al*, 2005). Elevated levels of modified nucleosides have been found in urine from patients with leukaemia and lymphoma (Rasmuson and Björk, 1995), cancer of the lung (McEntire *et al*, 1989), oesophagus (Masuda *et al*, 1993), breast (Rasmuson *et al*, 1987; Nakano *et al*, 1993; Sasco *et al*, 1996; Zheng *et al*, 2005), renal cell carcinoma (Koshida *et al*, 1985), ovarian cancer (Oerlemans and Lange, 1986), liver cancer (Yang *et al*, 2004), tumours of the bladder (Kvist *et al*, 1993), colon cancer (Holstege *et al*, 1986) and Hodgkin's disease (Martinow *et al*, 1998). One possible cause is the higher turnover rate of tRNA in tumour tissues than that in normal counterparts as demonstrated by Borek *et al* (1977).

For the quantification and identification of these nucleosides, immunoassays (Masuda *et al*, 1993; Ishiwata *et al*, 1995; Sasco *et al*, 1996), capillary electrophoresis (Yang *et al*, 2004; Zheng *et al*, 2005), column-switching or precolumn methodology (Boos *et al*, 1988) as well as high-performance liquid chromatography/mass spectrometry (Dudley *et al*, 2004) have all been applied. However, when a broad spectrum of modified nucleosides has to be determined, the preferred method is high-performance liquid chromatography (HPLC) (Lakings *et al*, 1977; Koshida *et al*, 1985; Oerlemans and Lange, 1986; Sander *et al*, 1986; Rasmuson *et al*, 1987; McEntire *et al*, 1989; Gehrke and Kuo, 1990; Schöch *et al*, 1990a, 1990b; Kvist *et al*, 1993; Nakano *et al*, 1993; Topp *et al*, 1993; Rasmuson and Björk, 1995; Martinow *et al*, 1998; Xu *et al*, 2000; Yang *et al*, 2004).

Approximately, 20 out of the now-known more than 90 urinary metabolites have frequently been used as diagnostic markers. Elevated concentrations of the following parameters have been suggested as possible markers: pseudouridine (Psi), 1-methyladenosine (m1A), 1-methylguanosine (m1G), 2-methylguanosine (m2G), 1-methylinosine (m1I), 2-pyridone-5-carboxamide-N1-ribofuranoside (PCNR) (1–24). The studies have shown that modified RNA molecules are metabolised, but not reincorporated into tRNA and excreted in the urine. Modified purines and pyrimidines, for example, pseudouridine, 5,6-dihydrouridine, N2,N2-dimethylguanosine and N6-threoninocarbonyladenosine, which are found only in tRNA, are quantitatively excreted in human urine (Borek *et al*, 1977; Sander *et al*, 1986; Schöch *et al*, 1990a; Topp *et al*, 1993). Consequently, all urine contains some modified nucleosides and the levels of modified nucleosides in urine reflect RNA degradation in the organism. However, all RNAs, especially tRNA, from neoplastic tissue have a much more rapid turnover rate than that of the corresponding healthy tissue (Borek *et al*, 1977). In addition, every tumour examined contains hyperactive tRNA methyltransferases (Lakings *et al*, 1977). There is strong evidence for the participation of other body cells in the release of modified nucleosides possibly due to tumour–host metabolic interactions (Borek *et al*, 1977). The concentrations of modified nucleosides in urine, primarily degradation products of tRNA, have been regarded as potential indicators for the whole-body turnover of RNAs, suggesting them as possible markers for malignant diseases (Sander *et al*, 1986; Schöch *et al*, 1990a, 1990b; Nakano *et al*, 1993; Topp *et al*, 1993). Sasco *et al* (1996) evaluated the prognostic significance of six urinary modified nucleosides in 68 breast cancer patients and found that excretion of 1-methylinosine and 1-methyladenosine was even further increased in patients with more advanced disease.

Nucleosides are an important class of metabolites and have the potential roles of serving as tumour markers. To further explore the usefulness of urinary nucleosides as tumour markers, we optimised the fractionation method and multivariable data analysis technique.

Developing a new method for fractionating of the nucleoside fraction using the Baker solid-phase extraction (SPE) system (Baker, 1997) with 3 ml columns with octadecylsilane, we studied the urinary profiles of nucleosides in patients with malignant diseases and control subjects. Determining normal nucleoside levels from ‘normal’ turnover and catabolism of RNA in the urine of healthy control subjects is necessary to compare the levels in patients with malignant diseases. A data set of urinary nucleosides containing all the patients and healthy volunteers was obtained for the neural network analysis.

## MATERIALS AND METHODS

### Reagents

The following nucleoside standards were obtained from Sigma (St Louis, MO, USA): pseudouridine, uridine, cytidine, 1-methyladenosine, 5-methylcytidine, 7-methylguanosine, inosine, 1-methylinosine, 5-methyluridine, guanosine, xanthosine, 1,7-dimethylguanosine, 1-methylguanosine, 2-methylguanosine, N2,N2-dimethylguanine, adenosine, N6-methyladenosine and 3-methyluridine. Modified nucleosides were all of HPLC purity grade. Ammonium dihydrogen phosphate, methanol and acetonitrile were Baker-analysed HPLC-grade from Baker (Phillipsburgh, NJ, USA). Distilled, deionised water was obtained from a Milli Q plus purification system (Millipore, Bedford, MA, USA).

### Collection of urine samples and extraction of nucleosides

Patients were completely recruited in the Department of Surgery II of the Medical Faculty of the University of Leipzig. The experimental groups consisted of 55 patients (age from 17 to 85 years; mean age 56.9 years) with histologically diagnosed malignant tumours (group I: 26 breast, eight colon, three thyroidea, seven sarcomas, two melanomas, two bronchial carcinomas, three granulomas, two gynaecological, two others, and group II: 13 patients with benign growths - nine adenomas of thyroidea, two chondromas, one lipoma, one adenoma of parathyroidea). Diagnoses of cancer and benign tumours were made on the basis of usual clinical, laboratory and radiologic findings and were confirmed by histopathology. In the Department of Surgery II, the method of staging was used according to the tumour node metastasis system (Spiessl, 1993). Data on serum carcinoembryonic antigen (CEA), cancer antigen (CA) 15–3 and tissue polypeptide antigen (TPA) were provided by the same hospital. Table 1 shows the clinical characteristics of 55 cancer patients. The control group was selected from healthy volunteers (31 students and 10 blood donors) with no manifestation of disease. Their age ranged from 25 to 70 years (mean age 38.4 years). At the time of the study, all were in good health with none taking any medications.

The urine samples, from both healthy and tumour patients, collected were mainly, but not exclusively, of the first thing in the morning. They were all filtered immediately using a 0.2-*μ*m membrane cellulose acetate (Sartorius). The filtered urine samples were stored at −20°C until processed. Modified nucleosides keep stable for at least 2 months when stored at −20°C. Urine is generally filtered sterile, and thus the concentration of nucleosides are not altered biologically.

The urinary modified nucleosides were extracted on octadecylsilane (C18) columns (Baker) possessing a specific affinity for *cis-*hydroxyl groups. The columns were preconditioned with 1.0 ml acetonitrile, followed by 5.0 ml methanol and 3.0 ml aqua dest. The recovery of nucleosides was estimated from a 0.5 ml stock solution passed through the columns. All analyses of the urine are based on a 0.5 ml volume. The columns were eluted with 2.0 ml methanol and 1.0 ml acetonitrile. The samples were then evaporated to dryness at 50°C and dissolved in 0.5 ml distilled water. A 20 *μ*l sample volume was injected into a reversed phase (RP) HPLC and analysed.

### Nucleoside and creatinine determinations

The chromatographic method used for the RP liquid chromatographic separation of nucleosides was based on the one developed by Gehrke and Kuo (1990). The HPLC apparatus (Gynkotek, München, Germany) consisted of a Gynkotek 480 pump gradient, heated column compartment (Gynkotek), an injector and a UVD 320 photodiode array ultraviolet (UV) detector. Separation was achieved using an (250 × 4.6 mm ID) LC-18-S Supelcosil column with guard cartridges (2.1 × 4.6 mm ID) LC-18-S Supelosil (Sigma-Aldrich, Bellefonte, PA, USA). For the calibration, four different volumes of a standard solution of nucleosides or creatinine were mixed with 0.5 ml of the internal standard (3-methyluridine), were treated separately on the SPE columns like the urine samples and were determined at 210, 245, 254 and 280 nm. Peaks in urine samples were identified by comparing their chromatographic retentions and UV spectra with known reference nucleosides, based on diode-array UV/VIS technology.

The nucleoside-to-creatinine ratio was chosen to compare patients and control subjects and to consider the circadian rhythm, because a 24-h urine sample was not available. A substantial element of the procedure is the determination of nucleosides and creatinine on a simultaneous way.

To determine simultaneously modified nucleosides and creatinine, we introduced an analytical fractionation of the sterile urine samples before the HPLC was carried out. For separation of the nucleoside fraction, we developed a method using the Baker SPE system with 3 ml octadecylsilane (C 18) cartridges operating under reduced pressure. The repeatability and reproducibility of nucleoside retention time were evaluated on a ‘run-to-run’ basis. The relative standard deviation (RSD) of the retention times for each of 18 nucleosides was calculated from 10 runs. The recoveries, determined earlier using urine spiked with the stock solution, were from 79 to 102%. The reproducibility of the method including the extraction has been determined in six repetitive analyses using a same normal spontaneous urine sample from a healthy volunteer extracted on SPE columns and analysed with the RP–HPLC.

### Statistical analysis

The univariate statistics and the nature of the underlying distributions were determined for each nucleoside. Separate univariate analysis of the 18 variables by using nonparametric statistics was carried out using the Kolmogorov–Smirnov test, given the non-normal distribution of half of the parameters studied. The Wilcox rank sum (*U*-test) and the Mann–Whitney test were applied in the comparison analyses of the median using the SPSS statistical package (Bühl and Zöfel, 1997). A mean value of *P*<0.05 was accepted as significant. Cutoff level was the average mean concentrations+standard deviations of each modified nucleoside in the urine of the 41 healthy volunteers. To avoid misclassifications of false positives and false negatives, a neuronal network analysis was applied to these data. This analysis is based on the network model developed by Zhao *et al* (1998) and is also applied here. Sensitivity and specificity are calculated with the aid of Baye's theorem, according to Wagener (1984).

## RESULTS

The calibration curves, with a mean correlation coefficient of 0.99, were obtained for 18 nucleosides and creatinine at four channels.

Repeated runs demonstrate the accuracy and precision of the technique used (Table 2). The range of standard deviation (RSD) of the retention is 0.20–1.69. The precision of the method was determined by six repetitive analyses of a normal male urine sample. The results in Table 2 show that the method is quantitative and reproducible. High RSD values were observed for m6A, which was caused by the transmethylation of m1A to m6A during sample preparation.

**Figure 1:**
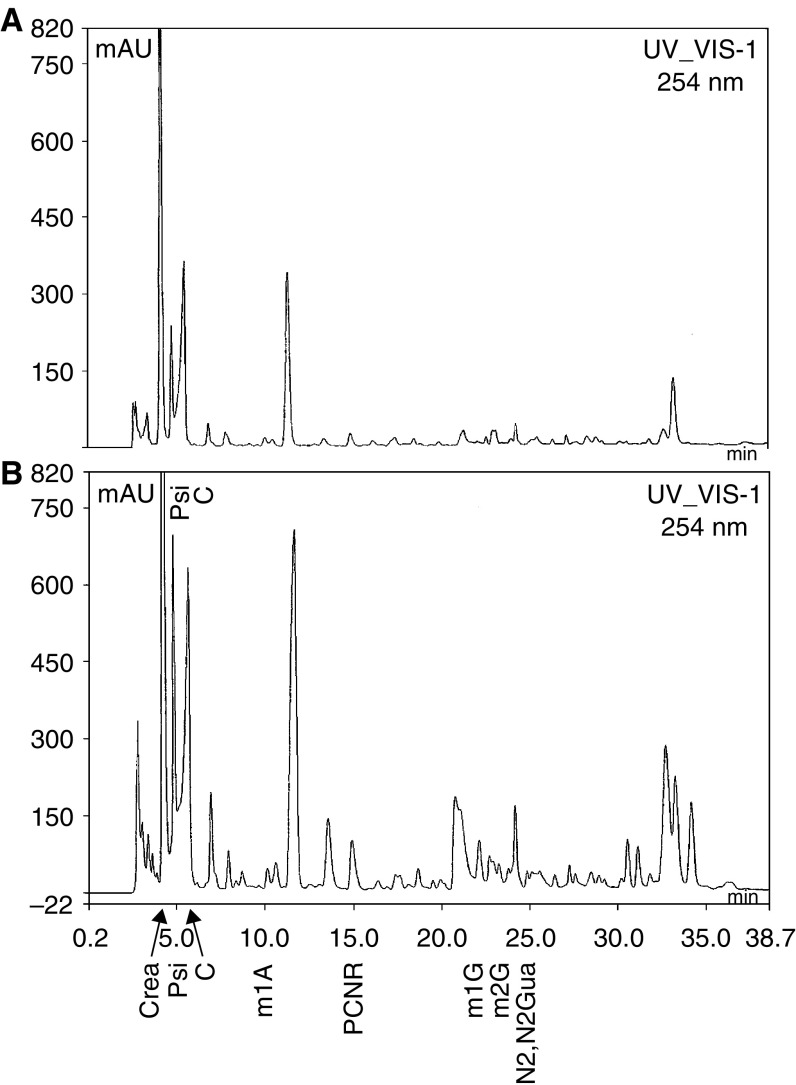
(**A**) Chromatograms of RP–HPLC separation of nucleosides in urine extract from a healthy person. Column: 250 × 4.6 mm Supelcosil LC-18S; mobile phase: gradient program; UV detection: 254 nm; peak identifications: Psi=pseudouridine, C=cytidine, m1A=1-methyladenosine, PCNR=2-pyridone-5-carboxamide-N1-ribofuranoside, M1G=1-methylguanosine, m2G=2-methylguanosine, N2,N2 Gua=N2,N2-dimethylguanine. (**B**) Chromatogram of RP–HPLC separation of nucleosides in urine extract from a cancer patient. Column: (250 × 4.6 mm) Supelcosil LC-18-S. All other chromatographic conditions were the same as for Figure 1A.

For example, representative chromatograms used for identification and quantification of nucleosides are given in Figures 1A and B showing the separation and resolution of nucleosides by using the chromatographic conditions described. Figure 1B is a chromatogram of urine of a malignant human and is representative of the HPLC method used in this study.

Urine from a total of 41 volunteers with no evidence of disease was analysed for 18 nucleosides. No significant difference was obtained between the levels of urinary nucleosides obtained from 30 men and 11 women (*P*>0.05). The mean age was 38.4 years (range was 25–70 years). No significant differences were found in the selected seven mean nucleoside values with respect to the age groups. Most of the nucleoside levels of this work were higher than the data given in literature.

In Table 3, the cutoff values of the modified nucleosides in the healthy group are shown serving as reference for the comparison with malignant tumour groups. Table 3 clearly shows that concentrations of cytidine, 1,7-dimethylguanosine, 1-methyladenosine, 1-methylguanosine, N2,N2-dimethylguanine, 2-methylguanosine, 5-methyluridine, 6-methyladenosine, PCNR and pseudouridine in urine of patients with cancer were elevated significantly.

**Figure 2:**
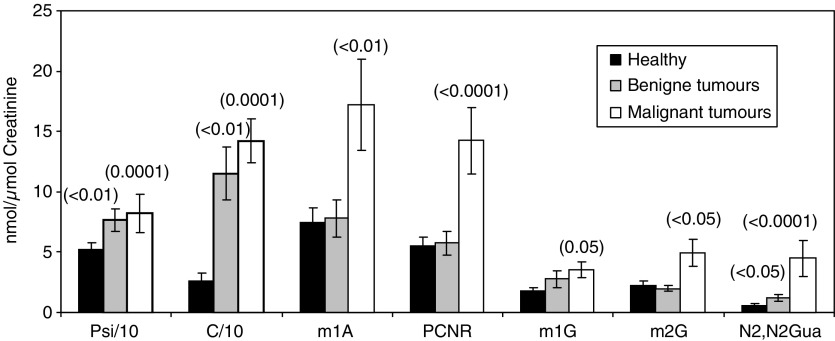
Mean excretion of modified nucleosides in urine from healthy volunteers (*n*=41) and patients with benign (*n*=13) and malignant tumours (*n*=55) (*P*-value for testing against ‘healthy’).

**Figure 3:**
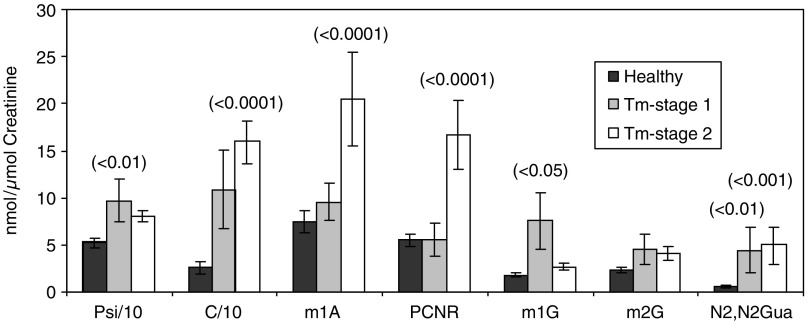
Mean excretion of modified nucleosides from healthy volunteers (*n*=41) and patients with malignant stages (stage 1=primary cancer (*n*=9); stage 2=patients have histological evidence of distant metastasis or recidivism (*n*=40)).

Our data presented in Figure 2 also revealed that the concentrations from patients with benign tumours were lower than those of cancer patients. Most modified nucleosides were found to be elevated, but the extent of the increase varied with each nucleoside. Levels of modified nucleosides excreted by patients with primary (stage 1) and of more advanced cancer (stage 2) are presented in Figure 3. The mean levels of cytidine, 1-methyladenosine, N2,N2-dimethylguanine and PCNR in patients with malignant cancer was higher than that with primary cancer. Increased numbers of peaks, which also have UV spectra like the spectra of nucleoside, were observed in samples of tumour patients in a more advanced stage. The fate of those missing nucleosides is yet unknown. It has to be assumed that these peaks are not tailings but real other nucleoside-like compounds.

The diagnostic value of a tumour marker depends on its sensitivity and specificity.

Significant elevations in concentrations of at least four of the following parameters cytidine, pseudouridine, 2-pyridone-5-carboxamide-N1-ribofuranoside, N2,N2-dimethylguanine, 1-methylguanosine, 2-methylguanosine, 1-methyladenosine and the total sum of 18 determined nucleosides indicate a sensitivity of 54% and a specificity of 98% (Table 4a). Assuming that three of the seven significant nucleoside parameters (given in Table 4b) should cross the threshold value, a sensitivity of 79% and a specificity of 85% could be achieved.

To avoid misclassifications of false positives and false negatives, a neuronal network analysis was applied to these data. The neuronal net was able to classify one unknown person as healthy or tumour patient. The result was then compared with the known health status of the person. This procedure was conducted for all of the 109 volunteers. From 68 tumour patients, 66 were classified correctly as tumour positive and two as tumour negative (false negative). From the 41 healthy persons, 35 were identified as healthy and six as false positive. The sensitivity and specificity were 97 and 85%, respectively.

The concentrations of CEA, CA 15–3 and TPA of these patients in serum and numbers of elevated nucleosides are given in Table 1. To distinguish subjects suffering from breast cancer and healthy subjects, the Baye's technique has been used (Wagener, 1984) to study the modified nucleosides resulting in a sensitivity of 76.9%. Carcinoembryonic antigen and CA 15–3 are conventionally used as tumour marker for breast cancer (cutoff level CEA=6 *μ*g l^−1^; CA 15–3=25 U ml^−1^). From Table 1, only three CEA levels in 24 breast patients and seven CA 15–3 levels in 24 breast cancer patients were higher than the cutoff value (sensitivity: CEA – 12.5% and CA 15–3 – 29.2%). Also, TPA has low diagnostic specificity (29.2%). In this study on women with breast cancer as well as in patients with different kinds of cancer, the modified nucleosides have a higher diagnostic sensitivity than CEA, CA 15–3 and TPA (Table 5).

## DISCUSSION

Modified nucleosides have been found in increased amounts in the urine of cancer patients (Figure 2). These RNA metabolites are not reincorporated for *de novo* nucleotide synthesis and, thus, quantitatively excreted in urine where they are measurable (Borek *et al*, 1977; Sander *et al*, 1986; Schöch *et al*, 1990a; Topp *et al*, 1993). Before the samples could be analysed by HPLC, a treatment is necessary to remove the proteins. Other authors reported on using phenyl boronate affinity column for nucleoside isolations (Koshida *et al*, 1985; Holstege *et al*, 1986; Oerlemans and Lange, 1986; Sander *et al*, 1986; Rasmuson *et al*, 1987; McEntire *et al*, 1989; Gehrke and Kuo, 1990; Schöch *et al*, 1990a; Kvist *et al*, 1993; Nakano *et al*, 1993; Topp *et al*, 1993; Rasmuson and Björk, 1995; Martinow *et al*, 1998; Xu *et al*, 2000; Yang *et al*, 2004) or using the Baker SPE system with aromatic sulphonic acid (Schöch *et al*, 1990b). These fractionation methods of nucleosides from urine were found to be too complex and time consuming to allow the analyses of large numbers of urine samples. We applied a new method using the Baker SPE system, which allowed the simultaneously chromatographic identification and quantification of creatinine and of nucleosides. In recent years, a number of methods based on HPLC have been reported, but little is known about simultaneous measurements of modified nucleosides and creatinine.

Random samples can be used when nucleoside levels are expressed relative to creatinine.

For the evaluation of the method, it was necessary to establish a reference range within the control group. Eighteen different modified nucleosides were measured in the urine of 41 healthy subjects (Table 3). The cutoff values of modified nucleosides are based on the values of the healthy group, serving as the reference for comparison of malignant and benign patient groups (*n*=55 and 13, respectively). In this study, the concentrations of 10 modified nucleosides were significantly elevated in patients with malignant diseases and six nucleosides in patients with benign tumours. The excretion of 1-methyladenosine, cytidine, N2,N2-dimethyguanine and PCNR were higher in patients, which have histological evidence of distant metastasis as compared to those in patients without metastasis, reflecting more advanced disease (Figure 3). Several modified nucleosides are always elevated in malignant diseases with varying levels. The following seven were the most significant ones: cytidine, N2,N2-dimethylguanine, PCNR, 2-methylguanosine, 1-methylguanosine, pseudouridine and 1-methyladenosine. These nucleosides have already been reported as useful biochemical ‘indicators’ in malignant diseases (Lakings *et al*, 1977; Koshida *et al*, 1985; Holstege *et al*, 1986; Oerlemans and Lange, 1986; Sander *et al*, 1986; Rasmuson *et al*, 1987; Boos *et al*, 1988; McEntire *et al*, 1989; Gehrke and Kuo, 1990; Schöch *et al*, 1990a, 1990b; Kvist *et al*, 1993; Nakano *et al*, 1993; Topp *et al*, 1993; Rasmuson and Björk, 1995; Martinow *et al*, 1998; Xu *et al*, 2000; Dudley *et al*, 2004; Yang *et al*, 2004; Zheng *et al*, 2005). Cytidine and uridine were found to be much more increased than reported in other publications. From Table 3, it can be observed that modified nucleosides (Psi, C, U, m1A) were more frequently higher than in the literature. We assume that these higher levels are due to the minimal sample manipulation during the new treatment method using SPE. It was also found that differences of nucleoside excretion exist among patients even with the same stage of tumour. This is illustrated in Figures 2 and 3, where m1G and m2G show different levels. This observation underlies the necessity to measure more than three or four nucleosides to evaluate the status of a subject. The sensitivity and specificity of the method applied here (four parameters elevated) were 54 and 98%, respectively, and, thus, fulfilled the requirements according to Fateh-Mogdaham and Stieber (1991). According to this, the diagnostic efficiency of a tumour test is to be fixed at a specificity of 95%, which results in a sensitivity of >50%. If the intention is to identify as many tumour patients as possible, the cutoff should be set low. Subsequently, an increase in the percentage of false-positive results has been observed, that is, a reduction in specificity. Based on the selection of three of the seven parameters above the threshold, a sensitivity of 79% (40 out of 55 patients are recognised as malignant) and specificity of 86% (five false positives) were achieved (Tables 1 and 5).

To analyse misclassifications of false positives and false negatives, an artificial neural network analysis (a pattern recognition tool) was applied. Application of this powerful method led to a reduced misclassification (only two out of 68 cases (97%) with tumours (malignant and benign)). Among healthy volunteers, five were misclassified as false positive (five out of 41; 84.5%). All in all, it can be stated that the artificial neural network technique leads to better results than principal component analysis (the Baye's theorem) for the classification of healthy persons and cancer patients based on nucleoside data (Zhao *et al*, 1998; Yang *et al*, 2002). The difference in misclassification between healthy and tumour patients cannot be explained at present. However, the authors assume that the threshold of nucleoside concentrations in the urine may not be exactly enough, that the number of nucleosides measured may not be large enough and/or that other specific combinations of nucleosides should be tested. This will be investigated further. However, McEntire *et al* (1989) measured 24 nucleosides in serum by HPLC and used a discriminate regression analysis (STEPDISC) classifying of lung cancer patients. Their sensitivity reached 84% with a specificity of 79%, showing that the misclassification rate did not get much better by increasing the number of nucleosides.

The concentrations of CEA, CA 15–3 and TPA in serum of the patients with breast cancer (Table 1a) show that the sensitivity of urinary nucleosides in breast cancer patients was 76.9% (20 out of 26 patients) by using the Baye's technique. Serum CEA and CA 15–3 are being used in clinics to diagnose breast cancer (cutoff level: CEA=6 *μ*g l^−1^; CA 15–3=25 U l^−1^). Table 1 shows a sensitivity of CEA and CA 15–3 as 12.5 and 29.2%, so the urinary nucleosides had higher diagnostic sensitivity than serum CEA and CA 15–3. In another study on women with breast cancer, the modified nucleosides had a higher diagnostic sensitivity than CEA and CA 15–3 (Rasmuson *et al*, 1987; Xu *et al*, 2000; Zheng *et al*, 2005). This is of particular interest, because breast cancer is the major cancer among women all over the world, and the used markers have low diagnostic sensitivity. Also, TPA in our study, which has been used as marker for breast cancer, has low diagnostic specificity (29.2%).

Further studies will be necessary to evaluate the usefulness of urinary nucleosides in differentiating cancer from other diseases. Attention may have to be paid to conditions influencing RNA catabolism other than those occurring in malignancies, for example, endocrine abnormalities, alcoholism, infections and renal dysfunction. This may increase the number of false positives.

In summary, we report on a sensitive HPLC method for the simultaneous determination of a broad spectrum of modified nucleosides and creatinine in one urine sample. The SPE fractionation method proposed here is simple and time sparing. This new preparation requires no extensive purification of the urine samples and no extensive preparation of columns, and requires small sample volume (0.5 ml) and an elution time of only 60 min. Urinary modified nucleosides cytidine, N2,N2-dimethylguanine, PCNR, 2-methylguanosine, 1-methylguanosine, pseudouridine and 1-methyladenosine were found to be a possible marker for cancer. In such a multicomponent alteration of the nucleoside levels, a pattern recognition method could reveal more information on the distinctions between healthy individuals and cancer patients than the evaluations of the single components. Compared with the Baye's theorem, classification by artificial neural networks is more satisfactory, and it can hopefully be used as a powerful tool for decisions in the tumour diagnosis. Simultaneous determination of modified nucleosides and creatinine is particularly advantageous in noninvasive diagnostic procedures.

## Figures and Tables

**Table 1:** Clinical characteristics of (a) breast cancer patients and (b) different types of cancer

**Cancer**	**Patient no.**	**Age**	**Sex**	**Cancer stage**	**Metastasis stage**	**CEA^a^ (*μ*g l^−1^)**	**CA 15-3^b^ (U l^−1^)**	**TPA^c^ (U l^−1^)**	**Number of elevated nucleosides**
(a)									
Breast cancer	1113	55	F	T4N1M*x*	Tms2	3.1	92.6	110.0	3
	1164	61	F	T2N1M*x*	Tms2	1.4	23.6	33.0	6
	1165	57	F	T3N1M0	Tms2	<1.0	85.6	222.0	1
	1166	53	F	T*x*N*x*M1	Tms2	<1.0	22.8	38.0	6
	1167	55	F	T2N1M*x*	Tms2	1.6	17.1	36.0	2
	1169	50	F	T2N1M1	Tms2	62.7	83.4	273.0	4
	1172	52	F	T4N0M0	Tms2	1.3	6.8	44.0	6
	1187	54	F	T2N1M0	Tms2	<1.0	5.2	8.0	3
	1193	41	F	T1N0M*x*	Tms2	2.4	12.2	30.0	3
	1199	78	F	T4N1M0	Tms1	1.8	20.9	67.0	5
	1204	52	F	T2N0M0	Tms1	2.6	10.1	15.0	5
	1209	59	F	T1N1M*x*	Tms2	<1.0	8.7	21.0	2
	1213	69	F	T2N1M*x*	Tms2	1.7	29.4	40.0	4
	1214	57	F	T4N*x*M*x*	Tms2	1.5	9.0	915.0	4
	1215	75	F	T4N1M1	Tms2	1.4	24.6	80.0	6
	1219	63	F	T2N*x*M0	Tms2				4
	1231	52	F	T4N0M0	Tms1	<1.0	<5.0	29.0	6
	1240	47	F	T4N1M1	Tms2	16.4	137.3	450.0	2
	1260	74	F	T0N*x*M1	Tms2	1.2	9.8	66.0	4
	1272	71	F	T*x*N*x*M1	Tms2	30.2	37.2	120.0	3
	1295	66	F	T1N*x*M*x*	Tms2	2.6	16.0	51.0	3
	1299	85	F	T2N0M0	Tms1				3
	1303	82	F	T1N1M0	Tms2	1.5	14.9	63.0	7
	1312	56	F	T2N0M0	Tms2	1.9	12.1	17.0	8
	1324	74	F	T1 N0M0	Tms1	1.3	25.2	72.0	1
	1334	37	F	T4N1M0	Tms2	<0.1	13.41	106.0	2

(b)									
Colon Ca.	1112	62	M	T3N2M1	Tms2	48.3		300.0	6
	1188	41	F	T*x*N0M0	Tms2	140.0			2
	1203	72	F	NA	Tms2	8.9		8.0	5
	1205	65	F	T3N0M1	Tms2				3
	1275	57	M	T3N0M0	Tms1	<1.0			1
	1267	41	M	T4N2M1	Tms2	5.0			2
	1279	50	M	T4N0M0	Tms2				5
	1296	44	F	T4N0M0	Tms1				3

Thyreoidea	1202	61	M	T4N1M1	Tms2				7
	1250	64	F	T2N1M1	Tms2	140.0			7
	1302	70	F	T0N0M1	Tms2				4

Sarkoma	1184	74	F	T2N0M0	Tms2				5
	1206	50	M	NA					5
	1218	83	M	T2N0M0	Tms1				2
	1263	50	F	T4N0M1	Tms2	<0.1	11.2	31.0	0
	1278	52	M	T2N*x*M*x*	Tms2				1
	1289	49	F	T2N*x*M1	Tms2	<1.0	12.6		3
	1298	17	M	T M*x*	Tms1				1

Melanoma	1220	62	M	T3N2M1	Tms2				4
	1287	85	F	NA	Tms2				6

Bronchial-Ca	1178	65	M	T4N3M1	Tms2				3
	1236	83	M	T2N1M1	Tms2	6.5	2.4		4

Granuloma	1168	53	M	Stad. IV a					0
	1180	24	F	NA					1
	1235	75	M	NA					3

Gynaecol. Ca	1297	33	F	T*x*N*x*M1	Tms2	<1.0	12.1		0
	1300	59	F	T*x*N*x*M1	Tms2	2.2	10.2	73.0	6

Other	1268	71	F	T4N0M0	Tms2	2.6			5
	1237	37	M						6
Average		58.6				4.33	10.1	77.0	4
s.d.		15.0				1.54	3.4		

CA=cancer antigen; CEA=carcinoembryonic antigen; F=female; M=male; s.d.=standard deviation; TPA=tissue polypeptide antigen.

Tms1 (tumour stage 1): patients have primary cancer but no evidence of distant metastasis.

Tms2 (tumour stage 2): patients have histological evidence of distant metastasis.

a Serum CEA normal: <6 *μ*g l^−1^.

b CA 15–3 normal: <25 U ml^−1^.

c TPA normal: <90 U ml^−1^.

**Table 2:** Analytical characteristics of RP–HPLC and RSD, in series determined in a normal urine sample

**Nucleoside**	**Abbreviation**	**Retention time mean**	**s.d.**	**RSD^a^ (%)**	**Linear range (mmol l^−1^)**	**Average (nmol μmol^−1^** **creatinine)**	**s.d. 2.72**	**RSD^b^ (%)**
Pseudouridine	Psi	4.96	0.06	1.21	0.010–0.200	73.36	3.968	5.41
Cytidine	C	6.30	0.08	1.27	0.010–0.200	42.41	2.969	7.00
1-methyladenosine	m1A	10.81	0.16	1.48	0.020–0.080	8.40	1.203	14.32
2-pyridone-5-carboxamide-N-1-ribofuranoside	PCNR	14.51	0.11	0.59	0.010–0.080	8.74	0.813	9.09
1-methylguanosine	m1G	23.61	0.09	0.38	0.010–0.050	2.47	0.524	21.21
2-methylguanosine	m2G	24.43	0.17	0.70	0.010–0.050	4.73	1.048	22.16
N2,N2-dimethylguanosine	N2,N2Gua	24.90	0.05	0.20	0.010–0.050	0.755	0.131	17.35
adenosine	A	25.37	0.21	0.83	0.010–0.050	4.74	1.396	29.45
1-methylinosine	m1I	23.20	0.13	0.56	0.010–0.050	5.80	1.158	19.96
3-methylcytidine	m3C	9.42	0.07	0.74	0.010–0.050	3.13	0.211	6.74
5-methylcytidine	m5C	12.18	0.19	1.56	0.010–0.050	12.53	1.944	15.51
5-methyluridine	m5U	18.50	0.08	0.43	0.010–0.200	15.72	4.924	31.32
1,7-dimethylguanosine	m1,7G	22.67	0.17	0.75	0.050–0.500	16.03	1.106	6.9

Inosine	I	17.69	0.14	0.79	0.010–0.080	6.42	0.786	12.24
6-methyladenosine	m6A	32.62	0.28	0.86	0.010–0.050	1.12	0.353	31.52
7-methylguanosine	m7G	16.96	1.00	1.00	0.010–0.050	2.08	0.278	13.36
uridine	U	8.85	0.15	1.69	0.010–0.100	6.05	0.200	3.31
Xanthosine	X	20.86	0.13	0.62	0.010–0.100	25.08	3.880	15.52

HPLC=high-performance liquid chromatography; PCNR=2-pyridone-5-carboxamide-N1-ribofuranoside; Psi=pseudouridine; RP=reversed phase; RSD^a,b^ (%)=relative standard deviation; s.d.=standard deviation.

**Table 3:** Nucleoside/creatinine ratios in urine of healthy persons and patients with malignant tumours

	**Healthy persons (*n*=41)**				**Malignant patients (*n*=55)**			
**Nucleoside (nmol *μ*mol^−1^)**	**Median**	**95% percentile**	**Mean**	**Cutoff level**	**Median**	**95% percentile**	**Mean**	**Significance**
Psi	43.23	114.515	52.203	88.008	66.598	185.384	81.927	<0.0001
C	6.012	136.383	26.118	67.89	128.66	426.782	142.181	<0.0001
m1A	5.121	23.356	7.491	14.622	11.58	52.578	17.2	<0.01
PCNR	5.268	16.966	5.508	9.602	8.31	57.557	14.232	<0.0001
m1G	1.616	4.876	1.808	3.296	1.928	12.072	3.536	<0.05
m2G	1.935	7.339	2.277	4.173	2.205	20.392	4.942	<0.05
N2,N2Gua	0.231	2.312	0.556	1.264	1.925	23.705	4.487	<0.0001
A	3.666	18.54	6.558	14.395	2.991	65.264	12.239	NS
m1I	4.362	12.312	5.223	9.23	4.388	75.247	14.63	NS
m3C	3.56	38.463	9.446	22.083	5.964	277.198	31.815	NS
m5C	20.145	54.394	20.408	37.275	24.123	128.998	32.135	NS
m5U	2.748	19.394	4.729	10.119	3.233	44.816	8.241	0.05
m1,7G	1.566	132.683	16.685	56.019	20.747	207.094	45.575	0.001
I	2.875	23.776	6.834	15.991	3.178	18.492	5.166	NS
m6A	0.445	4.05	0.894	2.57	1.189	6.05	1.892	<0.001
m7G	1.365	6.624	1.943	3.574	2.279	23.62	4.684	NS
U	3.376	55.619	7.216	23.087	2.018	63.453	10.819	NS
X	12.615	207.173	27.376	90.301	5.411	303.906	28.63	NS

NS=no significance; PCNR=2-pyridone-5-carboxamide-N1-ribofuranoside; Psi=pseudouridine.

**Table 4:**
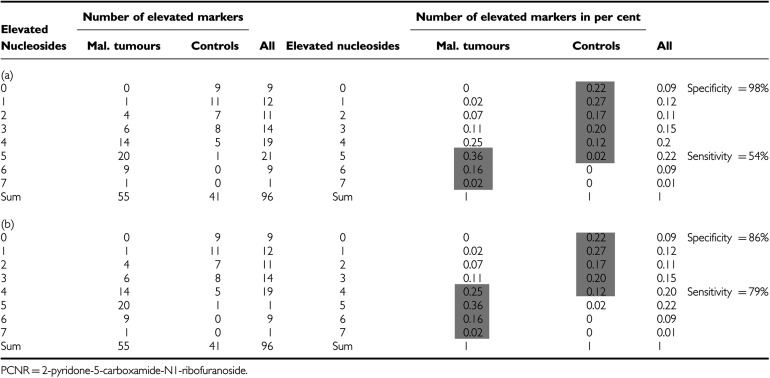
Discrimination between ‘healthy subjects’ and ‘tumour patients’ with the parameters (C, m1A, PCNR, m1G, M2G, N2,N2 G and sum of 18 determined nucleosides) as tumour markers. False negative/positive depending on elevated nucleosides

**Table 5:** Diagnostic positive ratio of cancer patients based on traditional biomarkers and on urinary nucleosides (sensitivity)

		**Traditional biomarkers**			
		**CEA**	**CA 15-3**	**TPA**	**Urinary nucleosides**
Breast cancer	*n*/*N*	3/24	7/24	7/24	20/26
	Sensitivity (%)	12.5	29.2	29.2	76.9

All kinds of cancer	*n*/*N*	8/35	7/31	8/28	40/55
	Sensitivity (%)	22.9	22	28.6	72.7

CA=cancer antigen; CEA=carcinoembryonic antigen; TPA=tissue polypeptide antigen.
